# The use of helical tomotherapy in the treatment of early stage breast cancer: indications, tolerance, efficacy—a single center experience

**DOI:** 10.18632/oncotarget.25286

**Published:** 2018-05-04

**Authors:** Alexandre Arsene-Henry, Jean-Philippe Foy, Magalie Robilliard, Hao-Ping Xu, Louis Bazire, Dominique Peurien, Philip Poortmans, Alain Fourquet, Youlia M. Kirova

**Affiliations:** ^1^ Department of Radiation Oncology, Institut Curie, Paris, France; ^2^ University Claude Bernard Lyon 1, INSERM 1052, CNRS 5286, Cancer Research Center of Lyon, Lyon, France; ^3^ Department of Radiation Oncology, Ruijin Hospital, Shanghai, China

**Keywords:** helical tomotherapy, early stage breast cancer, indications, tolerance, efficacy

## Abstract

**Purpose:**

to evaluate our experience in terms of local control, survival, adverse effects in patients treated by adjuvant helical tomotherapy (HT) for breast cancer (BC).

**Results:**

We studied 179 consecutive patients with 194 treated breasts with adjuvant HT. Median follow-up was 38.1 months. Median age was 53 years. Chemotherapy was administered to 83% of patients. All 133 hormone receptor positive tumours received hormonal therapy. As concurrent treatment, apart from trastuzumab monotherapy, 6 patients received systemic therapy concomitant to RT. The HT was generally well tolerated with mostly grade 1 and 2 skin reactions and esophagitis. Only 3% grade III early skin reactions. At last follow-up, there were 2 local recurrences, 1 regional lymph node (LN) recurrence and 6 with metastatic progression. The 5-year progression-free survival was 90.5% (95% CI 84.2–97.3).

**Materials and Methods:**

A retrospective study of all patients treated by HT between 2009 and 2015 was done. Patients excluded were those with: breast implants, advanced or metastatic BC, recurrent disease. All patients received breast+/-boost or chest wall irradiation and most received with LN irradiation. Dose constraints for organs at risk were defined using optimization scale developed in our Department. Evaluation of early and late toxicity was done using Common Terminology Adverse Criteria Events v.4.0.

**Conclusions:**

HT can be used for a well selected group of breast cancer as bilateral tumours, complex anatomy and target volumes where the conventional radiation therapy techniques cannot ensure an optimal dose distribution. Longer follow-up is necessary to confirm and validate these results.

## INTRODUCTION

Breast cancer is the most common malignant tumour in women worldwide [1]. Postoperative radiation therapy (RT) is part of standard treatment after surgery, either mastectomy or breast- conserving surgery. In particular, postoperative RT improves local control and disease-free survival and decreases mortality [2, 3]. A meta-analysis of several randomized trials demonstrated the benefit of internal mammary (IM), supraclavicular and infraclavicular lymph node irradiation in patients with axillary lymph node invasion or at high risk of recurrence [4].

However, RT can induce early and late adverse effects, including cosmetic sequelae and impaired quality of life [5]. It can also induce pulmonary and cardiac toxicity [6, 7]. Conventional breast or chest wall RT is based on two opposing tangential beams, resulting in high dose heterogeneity [8]. More recently, various techniques to optimize dose homogeneity, including intensity-modulated RT (IMRT), have been shown to be superior in terms of target volume coverage, and organ-at-risk (OAR) sparing [8–11]. This dosimetric optimization allows a reduction of RT-related adverse effects [12–15], while local control and survival appear to be similar [13, 16].

Rotational IMRT has been developed more recently, in the form of helical tomotherapy (HT) or volumetric-modulated arc therapy (VMAT). Dosimetric studies have shown that these techniques improve the target volume coverage and dose distribution homogeneity and can decrease the high dose to OAR, especially in the context of irradiation of complex volumes [17–21].

The purpose of this study was to evaluate local control, survival and adverse effects in patients treated by HT for non-metastatic breast cancer.

## RESULTS

Between 2009 and 2015, a total of 274 patients were treated for breast cancer by HT. Ninety-five patients were excluded from the study: 59 patients with metastatic disease, 9 patients with locally advanced tumour, 15 patients with breast prosthesis, 10 patients treated for recurrent disease and 2 patients with regional lymph node involvement with no known primary. Finally, 179 patients treated by postoperative HT for non-metastatic breast cancer were included in our study. Fifteen of these patients had a bilateral cancer, resulting in a total of 194 treated breasts.

### Patients and tumours characteristics (Table 1)

Median follow-up was 38.1 months (range: 7.4–78.2). The median age of the patients was 53 years (range: 25–76 years), of them only 25 patients (14%) were younger than 40. The characteristics of the patients included in the study are presented in Table 1A. The majority of patients received RT to one breast (*n* = 140), and 24 patients received chest wall RT. Right and left sides were treated with equal frequency. Fifty-three patients had a history of at least one cardiovascular disease and 18 patients had a history of lung disease.

Table 1Patients’ and tumour characteristics

**Table 1A:** Patients’ characteristics (*n* = 179)

Characteristics	*n*	%
Median age (range)	53 (25–76)	
Median BMI (range)	24.9 (16.3–53.4)	
Tobacco		
No	148	83
Yes	27	15
Unknown	4	2
Breast cup size		
A	10	5
B	48	25
C	51	26
≥ D	44	23
Unknown	41	21
Localisation		
Right breast	72	40
Left breast	68	38
Left chest wall	12	7
Right chest wall	12	7
Bilateral	15	8
History of CV disease		
AHT	28	16
Dyslipidaemia	25	14
Diabetes	8	4
Phlebitis	8	4
Arrhythmia	2	1
Myocardial infarction	1	1
Other	5	3
History of pulmonary disease		
Asthma	14	8
Chronic bronchitis	4	2
Other	1	1

*Abbreviations*: BMI = Body mass index; AHT = arterial hypertension; CV = cardiovascular.

**Table 1B:** Tumour characteristics (*n* = 194)

Characteristics	*n*	%
Quadrant		
External	83	43
Internal	77	40
Central	29	15
Unknown	5	2
Histology		
Invasive ductal carcinoma	162	85
Invasive lobular carcinoma	15	8
Carcinoma *In situ*	6	5
Mixed (Ductal and lobular)	5	3
Other	3	2
Clinical tumour stage		
cT1	102	53
cT2	59	30
cT3	24	12
cT4	6	3
Unknown	3	2
Clinical nodal stage		
cN0	93	48
cN1	80	41
cN2	1	1
cN3	4	2
Unknown	16	8
HER 2		
Yes	25	13
No	163	84
Unknown	6	3
Triple negative		
Yes	33	17
No	155	80
Unknown	6	3
HR+		
Yes	147	76
No	41	21
Unknown	6	3
SBR grade		
Low (I)	17	9
Intermediate (II)	78	40
High (III)	92	47
Unknown	7	4

*Abbreviations:* HER 2 = human epidermal growth factor receptor 2; HR+ = Hormone receptor positive; SBR = Scarff-Bloom-Richardson.

Tumours were nonspecific invasive carcinomas in 85% of cases, 5 patients had carcinoma *in situ*, 47% of tumours were grade 3, about one-half of patients had no clinical lymph node involvement and 33 patients had triple-negative tumours. Tumours characteristics are presented in Table 1B.

### Surgery

All patients were operated on, by breast-conserving surgery in 84% of cases and by mastectomy in the remaining cases. Axillary lymph node surgery consisted of immediate axillary lymph node dissection in 45% of cases, sentinel node procedure in 30% of cases and axillary lymph node dissection following a positive sentinel node in 23% of cases. No axillary lymph node surgery was performed in 3 patients.

### Systemic therapy

Chemotherapy was administered to 83% of patients, in the adjuvant setting in 61% of patients and in the primary setting in 39% of patients. Most patients (91%) received an anthracycline-based chemotherapy protocol followed by a taxane. Four of the 25 patients with an HER+ tumour did not receive trastuzumab. Almost three-quarters of the population (*n* = 133) received hormonal therapy for HR+ tumours. As concurrent treatment, apart from trastuzumab monotherapy, 6 patients received systemic therapy concomitant to RT, including FUN chemotherapy (5FU + vinorelbine) in 4 cases.

### Radiation therapy

Eighty-five per cent of patients received lymph node irradiation combined with breast or chest wall irradiation. Level II, III, IV and IM lymph nodes were irradiated in 57% of these patients; level IV and IM in 11% and all regional lymph nodes in 16%. No lymph node irradiation was performed in 15% of patients.

One hundred fifty-two patients (78%) received a boost dose to the tumour bed. In 83% of cases, the boost dose was delivered according to the simultaneous integrated boost (SIB) technique. Concomitant chemotherapy, mainly 5FU + vinorelbine, was administered in 3% of the cases.

The median duration of treatment was 46 days.

### Outcome (Figure 1)

**Figure 1:**
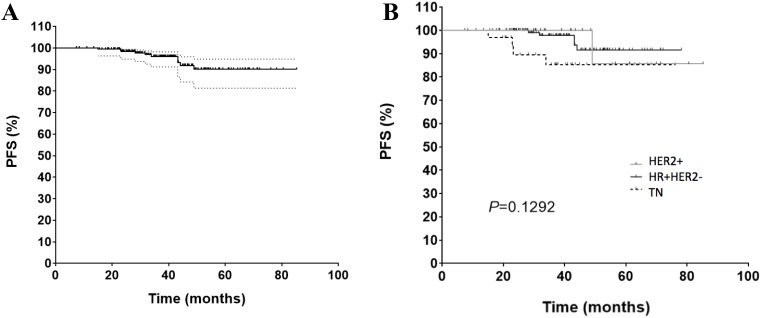
Progression-free survival (PFS) in (**A**) all the patients and (**B**) according to molecular profile. *Abbreviations*: PFS: Progression free survival; TN = triple negative; HER 2+ = human epidermal growth factor receptor 2 overexpressed; HR+ = Hormone receptor positive.

At last follow-up, there were 2 cases of local recurrence (1%), 1 case of regional lymph node recurrence and 6 cases of metastatic progression. The later consisted of 3 cases of lung metastases, 2 cases of bone metastases, 2 cases of liver metastases, 1 case of cerebromeningeal metastases and 1 case of choroidal metastases. Some patients presented with disease progression in multiple sites.

Three patients had died at the end of follow-up: 2 from breast cancer and one from metastatic malignant melanoma.

The 5-year progression-free survival (PFS) was 90.5% (95% CI 84.2–97.3) (Figure 1A). PFS by molecular subgroup was 83.4% (95% CI 69.6–99.9), 85.7% (95% CI 63.3–100) and 92.9% (95% CI 86–100) for triple- negative (TN), HER+ and HR+/HER2- subgroups, respectively (*p* = 0.13) (Figure 1B).

**Figure 2:**
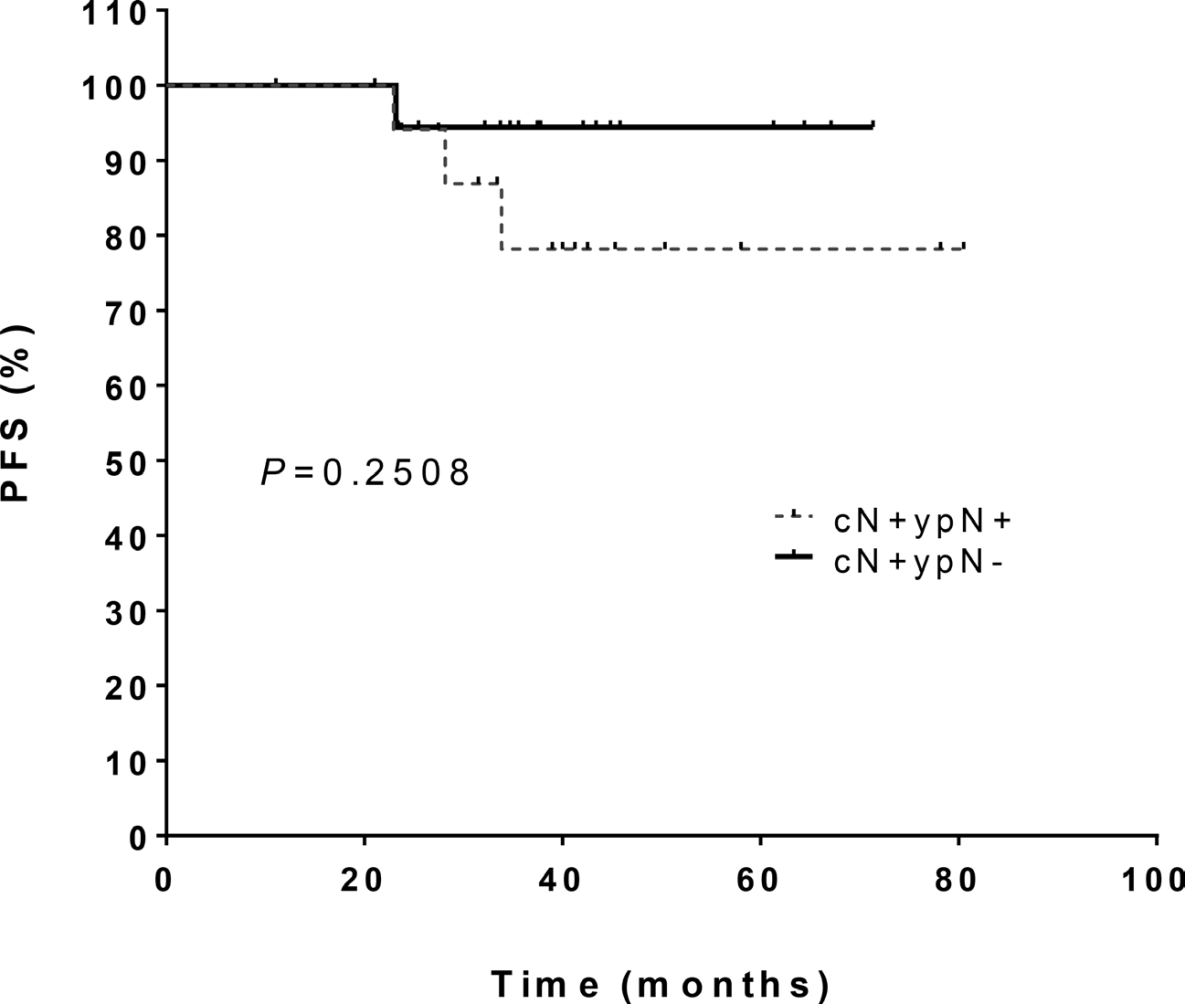
Progression-free survival in patients with initial clinically lymph node involvement (cN+) who received primary chemotherapy and who either had a complete remission lymph node status (ypN-) or maintained lymph node involvement (ypN+)

Patients with initial clinically lymph node involvement (cN+) lymph node involvement with negative lymph node status after primary chemotherapy had a 5-year PFS of 94.4% (95% CI 84.4–100). Patients with persistent lymph node involvement after primary chemotherapy had a 5-year PFS of 78.2% (95% CI 58.8–100) (*p* = 0.25) (Figure 2).

Four patients developed a second cancer after treatment of their breast cancer. A contralateral carcinoma *in situ* was diagnosed in one patient 1.6 years after completion of RT. One patient developed a left sacroiliac sarcoma, one patient developed a neuroendocrine tumour of the duodenum and another patient developed papillary thyroid carcinoma 1.3 years after completion of RT to the right chest wall and lymph node areas.

### Early toxicity (Table 2A and 2B)

Acute cutaneous toxicity consisted of radiation-induced dermatitis scored as grade 0 or 1 in 57% of patients, grade 2 in 40% of cases and grade 3 in 3% of cases. Gastrointestinal toxicity consisted of esophagitis in 18% of cases (16% of grade 1 and 2% of grade 2, but no grade 3). All patients experiencing acute esophagitis were irradiated to level IV and IM lymph nodes. Two patients experienced dry cough that resolved spontaneously during RT. Decreased left ventricular ejection function (from 62% to 50%) was observed during RT in 1 hypertensive patient, who had received anthracycline-based adjuvant chemotherapy in combination with trastuzumab prior to irradiation of the left breast with a boost dose and irradiation of the, level II, III, IV, IP and IM lymph nodes.

A high body mass index (BMI) (*p* < 0.0001) and a history of cardiovascular disease (*p* = 0.04), especially hypertension (HT) (*p* = 0.005), were associated with a significantly higher risk of acute cutaneous toxicity. Cup size and smoking did not significantly influence this risk. On multivariate analysis, only BMI was significantly associated with an increased risk of acute cutaneous toxicity. The results of these analyses are presented in Table 2A and 2B.

Table 2Toxicity

**Table 2A:** Univariate analysis of risk factors for acute and late skin toxicity

	Acute skin toxicity^*^			Late skin toxicity^*^		
	No (%)	Yes (%)	*P*-value	No (%)	Yes (%)	*P*-value
Age						
Median	51	55	0.1987	51	56	0.039
[min; max]	[25; 74]	[32; 76]		[25; 74]	[32; 76]	
BMI						
Median	23.5	27.3	< 0.0001	24.02	27.3	0.004
[min; max]	[16.3; 53.4]	[17.7; 44.5]		[16.3; 53.4]	[18.9; 51.3]	
Tabaco use						
Yes	13 (11.8)	17 (20.2)	0.159	20 (15.5)	7 (13.0)	0.819
No	94 (85.5)	65 (77.4)		106 (82.2)	45 (83.3)	
Unknown	3 (2.7)	2 (2.4)		3 (2.3)	2 (3.7)	
Breast cup size						
A-B-C	72 (65.5)	49 (58.3)	0.1134	89 (69.0)	28 (51.9)	0.018
D-E-F-G	20 (18.2)	25 (29.8)		24 (18.6)	19 (35.2)	
Unknown	18 (16.4)	10 (11.9)		16 (12.4)	7 (13.0)	
History of CV disease						
Yes	26 (23.6)	32 (38.1)	0.0397	38 (29.5)	18 (33.3)	0.725
No	83 (75.5)	52 (61.9)		90 (69.8)	36 (66.7)	
Unknown	1 (0.9)	0 (0.0)		1 (0.8)	0 (0.0)	
AHT						
Yes	10 (9.1)	21 (25.0)	0.005	19 (14.7)	11 (20.4)	0.385
No	99 (90.0)	63 (75.0)		109 (84.5)	43 (79.6)	
Unknown	1 (0.9)	0 (0.0)		1 (0.8)	0 (0.0)	
Diabetes						
Yes	4 (3.6)	5 (6.0)	0.5062	8 (6.2)	0 (0.0)	0.107
No	105 (95.5)	79 (94.0)		120 (93.0)	54 (100.0)	
Unknown	1 (0.9)	0 (0.0)		1 (0.8)	0 (0.0)	
Dyslipidaemia						
Yes	13 (11.8)	15 (17.9)	0.3036	18 (14.0)	10 (18.5)	0.514
No	96 (87.3)	69 (82.1)		110 (85.3)	44 (81.5)	
Unknown	1 (0.9)	0 (0.0)		1 (0.8)	0 (0.0)	

^*^For acute skin toxicity: “No” included grade 0 and 1; for late skin toxicity: “No” included only grade 0.

*Abbreviations:* BMI = Body mass index; AHT = arterial hypertension; CV = cardiovascular.

**Table 2B:** Multivariate analysis of risk factors for acute and late skin toxicity

	Acute skin toxicity			Late skin toxicity		
OR	95% CI	*P*-value	OR	95% CI	*P*-value	
Age	1.01	[0.98–1.04]	0.56	1.03	[0.99–1.06]	0.12
BMI	1.07	[1.01–1.15]	0.04	1.07	[1.00–1.14]	0.048
Breast cup size^*^	1.24	[0.55–2.74]	0.6	1.81	[0.81–4.03]	0.14
AHT	2.55	[0.87–8.15]	0.1	NA	NA	NA

^*^Breast cup size (A-B-C vs D-E-F-G)

*Abbreviations:* BMI = Body mass index; AHT = arterial hypertension; CV = cardiovascular; OR = odds ratio; CI = confidence interval; NA = not analysed.

**Table 2C:** Late toxicities (*n* = 194)

Late toxicity	Yes (%)	No (%)	Unknown (%)
Pulmonary	(0)	(100)	(0)
Cardiac	(0)	(100)	(0)
Cutaneous	60 (31)	130 (67)	4 (2)
	**Grade 1 (%)**	**Grade 2 (%)**	**Grade 3 (%)**
Hyperpigmentation	18 (9)	1 (1)	0
Breast oedema	14 (7)	1 (1)	0
Fibrosis	24 (12)	3 (2)	0
Telangiectasia	4 (6)	2 (1)	0
Breast pain	11 (6)	1 (1)	0

Of note, patients with high BMI (> 25 kg.m^-2^) have a higher risk of acute skin toxicity (grade 0 or 1 versus grade 2 or more) compared to patients with BMI < 25 kg.m^-2^, in a multivariate analysis including age, HT and cup size (OR = 4,1; 95% IC 1,97–8,83; *p* = 0,0002).

None of the risk factors for acute cutaneous toxicity studied was significantly associated with an increased risk of acute gastrointestinal toxicity.

### Late toxicity (Table 2)

Late toxicities are summarized in Table 2C. Late cutaneous toxicity was observed in 31% of patients. This toxicity was limited to grade 1 in the great majority of cases with hyperpigmentation in 9% of cases, fibrosis in 12% of cases, breast edema in 7% of cases, and telangiectasia in 6% of cases. Grade 2 late cutaneous toxicities were observed in 5% of cases, with no cases of grade 3 toxicity. Six per cent of patients experienced persistent grade 1 breast pain. No late cardiac or pulmonary toxicity was observed.

Advanced age (*p* = 0.04), higher BMI (*p* = 0.004), and large cup size (*p* = 0.02) were significantly associated with an increased risk of late cutaneous toxicity. In contrast, neither a history of cardiovascular disease nor smoking was significantly associated with an increased risk of late cutaneous toxicity. On multivariate analysis, only BMI was significantly associated with an increased risk of late cutaneous toxicity. The results of these analyses are presented in Table 2A and 2B.

### OAR and target volumes

Results concerning target volume coverage are previously reported [11]. HT ensures good coverage in the presence of complex volumes.

The results of dosimetric analysis in terms of OAR are presented in Table 3. In this study, the mean dose received by the heart, ipsilateral lung, contralateral lung and contralateral breast was 7 Gy, 13.5 Gy, 5 Gy and 3.8 Gy, respectively. The ipsilateral lung received a mean V30 equal to 9.6%. In contrast, the contralateral lung and breast received low doses with a mean V5 equal to 32% and 16.3%, respectively.

**Table 3:** Doses to organs at risk according to the irradiated area (Mean +/− SD)

	Heart	Homolateral lung				Controlateral lung		Controlateral breast	
	Mean dose (Gy)	Mean dose (Gy)	V20 (%)	V30 (%)	Mean dose (Gy)	V5 (%)	V20 (%)	Mean dose (Gy)	V5 (%)
Right breast	6.8 +/−1.3	13.8 +/− 1.9	20.8 +/− 5	9.3 +/− 3.8	4.4 +/− 1.3	31.9 +/− 11.4	0.5 +/−2.4	3.9 +/−1.1	16.3 +/−11.1
Left breast	6.9 +/−1.6	13 +/− 2.3	20.7 +/− 5.5	9.5 +/− 3.2	4.7 +/− 0.8	36 +/− 11.1	0.2 +/−0.3	3.9 +/− 0.8	16.7 +/− 9.2
Right chest wall	6.8 +/−1.0	14 +/−1.8	23 +/− 4.9	10.2 +/−2.8	4.0 +/− 0.5	27.9 +/− 6.7	0	3.6 +/− 0.6	15.5 +/− 7.3
Left chest wall	7.8 +/− 1.1	13.3 +/− 1.7	20.3 +/− 4.8	9.3 +/− 3.3	6.8 +/− 6.6	32.5 +/− 4.5	0.4 +/−0.5	3.9 +/− 0.7	16.6 +/− 9.0

*Abbreviations:* SD = Standard deviation; Vx = volume that received more than xGy.

## DISCUSSION

This largest with the longest follow-up study confirms that the HT is a well-tolerated treatment for breast cancer, with good local and distant disease control, especially in complex volumes (described above) when RT cannot be delivered via conventional (3D-CRT) techniques. This series represents the first large homogeneous single center experience in the use of helical tomotherapy in terms of efficacy and toxicity, as well as the practical proposal of adapted doses to OAR in these particular situations (bilateral cancers in 15% of patients, 85% of lymph node irradiation, high number of pectus excavatum). These complex volumes explain the higher doses to heart in comparison with the general recommendations [6]. The main limitation of this study is its retrospective nature and the period of follow-up of 38.1 months, which can be explained by the fact that HT was initially used for other tumour sites, but, after a number of years of experience, its indications have now been extended [18, 22]. Low level of complications was observed in terms of lung and heart toxicity. Progression-free survival in this study was very satisfactory, but these results must be interpreted cautiously in view of the relatively short median follow-up.

IMRT, especially helical HT, has been shown to improve target volume coverage, dose conformity, and dose homogeneity [8, 11, 19]. This dosimetric improvement can reduce acute and late toxicity [14, 23]. However, these techniques raise the issue of low-dose irradiation of a larger volume of healthy tissues and its possible long-term impact, particularly in terms of radiation-induced cancer [24, 25], especially as breast cancer patients have a long mean life expectancy [26]. In order to decrease this risk, we decided not to treat women under the age of 40.

Breast or chest wall irradiation by conventional techniques can lead to irradiation of a part of the heart and be associated with an increased risk of late cardiac toxicity depending on the mean dose to the heart [6]. Several approaches have been introduced to lower the radiation dose to the heart, including cardiac shielding, respiratory control and (volumetric) IMRT [11, 17, 19]. However, HT delivers low doses to a larger part of the heart, resulting in a higher mean dose. Longer follow-up of these patients is essential to evaluate the long-term impact of this low-dose irradiation. In the meantime, IMRT should be combined with respiratory control in selected patients [24]. HT allows a significant reduction of the dose received by the ipsilateral lung, but, due to its rotational nature, this technique induces low-dose irradiation of the contralateral lung, resulting in an increased mean dose received and V5% [11, 17]. Another limitation to the use of the HT in breast cancer is the low-dose irradiation of the contralateral breast, to a much lower extent observed with conventional techniques [27], raising the possibility of radiation-induced secondary cancers [25, 27–29]. Stovall et al. showed that women under the age of 40 who received a dose greater than 1 Gy to the contralateral breast had an increased long-term risk of developing a second primary breast cancer [29]. This excess risk was not observed in women > 40.

The simultaneous integrated boost (SIB) technique has already been applied to breast cancer RT [22, 30–32]. In combination with 3DCRT or IMRT, this technique improves dose conformity at the tumour bed, decreases the delivery of high doses and the dose to OAR compared to a sequential boost [22, 30–32]. SIB induces a reduction of treatment time and increased doses per fraction to the tumour bed, which could theoretically increase local control [33].

## MATERIALS AND METHODS

### Patients

A retrospective study was conducted in the Department of Radiation Oncology. All patients treated by HT between 2009 and 2015 for non-metastatic breast cancers were included in the study. Patients presenting with the following criteria were excluded: breast implants, advanced and metastatic breast cancer, recurrences. Data were collected until March 2017.

HT was used in specific cases in which conventional techniques were unsatisfactory in terms of target volume coverage and dose to OAR, most commonly corresponding to patients with unusual anatomy (pectus excavatum, narrow intermammary cleft), large breast volume, deeply seated IM lymph nodes, medial tumours with associated IM irradiation, bilateral cancer with lymph node irradiation. A high proportion of the patients’ population was referred by other radiotherapy departments because of the inability to treat the patients with conventional techniques and acceptable doses to OAR.

The following parameters were analyzed: patient and tumour characteristics, treatments received, early and late toxicities, local, regional and distant recurrences, and progression-free survival (PFS). PFS corresponds to the time between the end of RT and local, regional or distant disease progression. Patients had a clinical examination every week during radiation therapy and after, the follow-up consisted of every 4 months clinics in patients who received chemotherapy and every 6 months till the 5th year after the treatment, then once per year.

### Surgery

First-line breast-conserving treatment was performed whenever possible. Some patients received primary systemic treatment. Mastectomy was performed when breast-conserving surgery was not possible. Breast surgery included lymph node dissection in N+ patients, and sentinel lymph node biopsy in N-neg. patients, completed by axillary lymph node dissection in the case of positive sentinel node.

### Systemic therapy

The majority of patients received chemotherapy, mostly anthracycline-based chemotherapy followed by taxanes. When the tumour overexpressed the Human Epidermal Growth Factor-2 (HER-2) receptor, patients received trastuzumab for one year. Patients with tumours expressing hormone receptors received hormonal therapy for 5 years adapted to their menopausal status: Tamoxifen for premenopausal women and an aromatase inhibitor for postmenopausal women.

### Treatment planning CT scan

A CT scan (3 mm slices) was performed from the Tragus to L2/L3 without contrast agent using a Toshiba Aquilion LB scanner (Toshiba). Patients were placed in supine position with an AIO positioning system (ORFIT, Wijnegem Belgium) on a 5° inclined plane. An immobilization device was placed under the patient's knees. Both arms were positioned above the patient's head. A chin rest integrated in a heat-formed mask limited repositioning errors. Both breasts and the surgical scars were marked with radiopaque markers.

### Target volumes delineation

CT sections were transferred to the contouring system (Eclipse 3D version 13.6; Varian Medical Systems Inc., Palo Alto, USA). The breast/chest wall and lymph node clinical target volumes (CTV) were delineated according to our guidelines then ESTRO guidelines after the official publication [34]. The primary tumour bed was contoured according to previously described methods [35]. A 5 mm expansion around the CTV was performed to define the planning target volume (PTV). The PTV was cropped 3 mm under the skin.

### Prescription

The prescribed dose was 50 Gy in 25 fractions (2 Gy/fraction) to the breast/chest wall and lymph nodes. When a breast with boost was indicated, it was delivered either sequentially at a dose of 16 Gy in 8 fractions or, in the majority of cases, by a simultaneous integrated boost technique, which delivered 52.2 Gy in 29 fractions (1.8 Gy/fraction) to the breast and 63.8 Gy (2.2 Gy/fraction) to the tumour bed. The dose was restricted then to 50.4 Gy (1.74 Gy/fraction) to the lymph node areas. The objective was the homogenous cover of 95% of the PTV by > 95% isodose.

### Organs at risk: optimization of dosimetry and dose constraints

Fifty consecutive HT treatment plans to the breast or chest wall with lymph node irradiation, were used to calculate dose-volume histogram (DVH) values for each OAR (heart, ipsilateral lung, contralateral lung, contralateral breast and bone marrow). These dose values were classified in increasing order and divided into 4 classes (quartiles). Four quartiles of dose values were defined for each organ. *Q1* represents the maximum dose in the first quartile. Only 25% of treatment plans of the sample therefore presented a dose to the organ at risk of less than or equal to the *Q*1 value. *Q*2 represents the value of the median dose of the sample. *Q3* represents the maximum dose in the third quartile. Only 25% of treatment plans presented a higher dose than the *Q*3 value. *Q4* represents the maximum dose of the sample (Table 4).

**Table 4:** Dose to organs at risk treated with helical tomotherapy

**Heart**						
	Dmean (Gy)	Dmed (Gy)	V5 (%)	V10 (%)	V25 (%)	
Q1	< 6.4	< 5	< 48	< 17	< 0	
Q2	< 7.1	< 6	< 57	< 20	< 2	
Q3	< 8.5	< 7	< 69	< 25	< 3	
Q4	< 10.3	< 8	< 83	< 35	< 6	
**Ipsilateral lung**						
	Dmean (Gy)	Dmed (Gy)	V5 (%)	V20 (%)	V30 (%)	
Q1	< 11.9	< 7.7	< 67	< 17	< 7	
Q2	< 13	< 9.2	< 75	< 20	< 9	
Q3	< 14.6	< 10.6	< 88	< 23	< 13	
Q4	< 18	< 14.4	< 100	< 33	< 17	
**Contralateral breast**						
	Dmean (Gy)	Dmed (Gy)	V3 (%)	V5 (%)	V7 (%)	V10 (%)
Q1	< 3.3	< 2.8	< 44	< 8	< 2.3	< 0
Q2	< 3.6	< 3.2	< 59	< 14	< 3.3	< 0
Q3	< 3.9	< 3.3	< 64	< 17	< 6.2	< 1
Q4	< 5.7	< 4.3	< 89	< 37	< 19.7	< 9
**Contralateral lung**						
	Dmean (Gy)	Dmed (Gy)	V5 (%)	V7 (%)	V10 (%)	
Q1	< 4	< 3.6	< 29	< 9	< 1	
Q2	< 4.5	< 4.1	< 35	< 12	< 2	
Q3	< 4.8	< 4.5	< 40	< 16	< 4	
Q4	< 6.6	< 5.9	< 69	< 32	< 12	
**Spinal cord**						
	Dmean (Gy)	Dmed (Gy)	D max (Gy)			
Q1	< 5.6	< 3.1	< 23			
Q2	< 7.3	< 5	< 28			
Q3	< 8.8	< 6.5	< 34			
Q4	< 11.9	< 10.6	< 42			

*Abbreviations*: Q = quartile; Dmean = mean dose; Dmed = median dose; Dmax = maximum dose; Vx = volume that received more than xGy.

For all new treatment plans, the lower than *Q2* dose constraint is now applied to each organ-at-risk in order to obtain optimal and sufficient intensity modulation of the beam to comply with clinical constraints. These dose constraints were developed in our Department with aim to decrease the doses to OAR in patients with complex anatomy and/or volumes of irradiation.

### Helical tomotherapy treatment planning

CT scan and contoured volumes were transferred to the HT planning station (TomoTherapy HI-ART version 3.1.2.3; TomoTherapy Inc., Madison, United States). All treatment plans were calculated with a pitch of 0.286, a modulation factor initially set at 2.5 and a collimation of 2.5 cm.

Two fictitious volumes were created in the treatment planning system to limit the low doses delivered to healthy tissues. No irradiation was allowed when the accelerator passed over the contralateral hemi body or the patient's posterior surface.

### Patients’ follow-up and evaluation of toxicities

Patients were examined weekly during RT, then 4 to 6 months after the end of RT and then every 6 months, alternately by the medical oncologist (in the case of chemotherapy), surgeon/gynaecologist and radiation oncologist. Acute cutaneous, gastrointestinal, pulmonary and cardiac toxicities were evaluated retrospectively using Common Terminology Adverse Criteria Events v.4.0 [36].

Late toxicities were evaluated on the most recent consultation report and at least 6 months after completion of RT.

### Statistical methods

Statistical analysis was performed with R programming language and GraphPad Prism software version 6.00, (GraphPad software, Inc., San Diego, CA). The distributions of quantitative and qualitative variables were expressed by the mean and standard deviation (quantitative variables), or as a percentage (qualitative variables). Statistical analysis of qualitative variables was performed by Fisher's exact test. Mann-Whitney's nonparametric test was used to compare each continuous quantitative variable between the two groups. The association between clinical factors and gastrointestinal and cutaneous toxicity was tested on multivariate analysis using a logistic regression model. Specific progression-free survival (PFS) was defined as the interval between the end of RT and the date of the first disease-related event (local, regional or distant recurrence and cancer-related death). Survival curves were plotted by the Kaplan-Meier method and were compared by a log-rank test. A *p* value < 0.05 was considered to be statistically significant.

## CONCLUSIONS

HT can be used for a well selected group of breast cancer such as bilateral tumours, complex anatomy and target volumes where the conventional techniques cannot ensure an optimal dose distribution with good efficacy and tolerance. Longer follow-up is necessary to confirm and validate these results.
